# Phylogeny and Historical Biogeography of *Paphiopedilum* Pfitzer (Orchidaceae) Based on Nuclear and Plastid DNA

**DOI:** 10.3389/fpls.2020.00126

**Published:** 2020-02-27

**Authors:** Chi-Chu Tsai, Pei-Chun Liao, Ya-Zhu Ko, Chih-Hsiung Chen, Yu-Chung Chiang

**Affiliations:** ^1^ Kaohsiung District Agricultural Research and Extension Station, Pingtung, Taiwan; ^2^ Department of Biological Science and Technology, National Pingtung University of Science and Technology, Pingtung, Taiwan; ^3^ School of Life Science, National Taiwan Normal University, Taipei, Taiwan; ^4^ Department of Biological Sciences, National Sun Yat-sen University, Kaohsiung, Taiwan; ^5^ Department of Botany, National Museum of Natural Science, Taichung, Taiwan; ^6^ Department of Biomedical Science and Environment Biology, Kaohsiung Medical University, Kaohsiung, Taiwan

**Keywords:** *Paphiopedilum*, molecular phylogeny, biogeography, evolutionary trend, dispersal events

## Abstract

The phylogeny and biogeography of the genus *Paphiopedilum* were evaluated by using phylogenetic trees derived from analysis of nuclear ribosomal internal transcribed spacer (ITS) sequences, the plastid *trn*L intron, the *trn*L-F spacer, and the *atp*B-*rbc*L spacer. This genus was divided into three subgenera: *Parvisepalum*, *Brachypetalum*, and *Paphiopedilum*. Each of them is monophyletic with high bootstrap supports according to the highly resolved phylogenetic tree reconstructed by combined sequences. There are five sections within the subgenus *Paphiopedilum*, including *Coryopedilum*, *Pardalopetalum*, *Cochlopetalum*, *Paphiopedilum*, and *Barbata*. The subgenus *Parvisepalum* is phylogenetic basal, which suggesting that *Parvisepalum* is comprising more ancestral characters than other subgenera. The evolutionary trend of genus *Paphiopedilum* was deduced based on the maximum likelihood (ML) tree and Bayesian Evolutionary Analysis Sampling Trees (BEAST). Reconstruct Ancestral State in Phylogenies (RASP) analyses based on the combined sequence data. The biogeographic analysis indicates that *Paphiopedilum* species were firstly derived in Southern China and Southeast Asia, subsequently dispersed into the Southeast Asian archipelagoes. The subgenera *Paphiopedilum* was likely derived after these historical dispersals and vicariance events. Our research reveals the relevance of the differentiation of *Paphiopedilum* in Southeast Asia and geological history. Moreover, the biogeographic analysis explains that the significant evolutionary hotspots of these orchids in the Sundaland and Wallacea might be attributed to repeated migration and isolation events between the south-eastern Asia mainland and the Sunda Super Islands.

## Introduction

The orchid genus *Paphiopedilum* Pfitzer belongs to the subfamily Cypripedioideae Lindley. This subfamily has been considered a distinct lineage since Lindley (1840) separated them from other orchids based on the characteristic of having two separated fertile anthers [see (Cribb, 1998)]. This subfamily includes only five genera: *Cypripedium*, *Mexipedium*, *Paphiopedilum*, *Phragmipedium,* and *Selenipedium*. *Mexipedium* and *Selenipedium* are monotypic genera (Albert and Chase, 1992), which was a finding supported by ITS sequence analysis (Cox et al., 1997). These five genera are distributed in separate and restricted geographical ranges (Cribb, 1998). *Paphiopedilum* is distinguished from genera *Cypripedium* and *Selenipedium* by its conduplicate coriaceous leaves, as opposed to the plicate persistent leaves of the latter two genera. Furthermore, *Paphiopedilum* differs from *Phragmipedium* and *Mexipedium*, as they display imbricate sepal vernation, different chromosome base numbers and a unilocular ovary (Albert and Chase, 1992; Albert, 1994).

**Figure 1:**
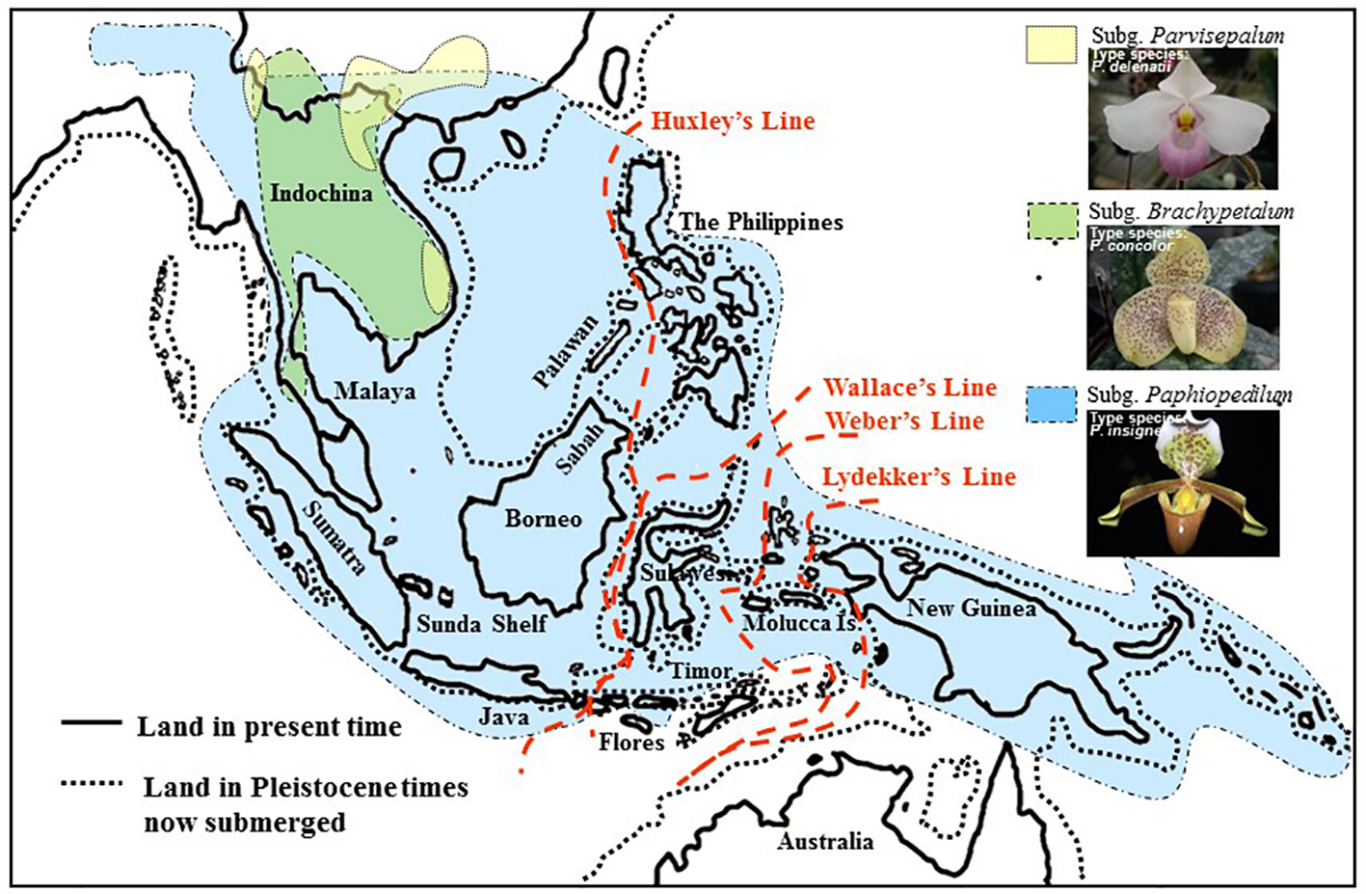
Map of the geographical distribution of *Paphiopedilum* based on the phylogeny of Cribb (1998). Comparison of Southeast Asian landmasses between the Pleistocene era and the present. During the Pleistocene, Indochina, Malaya, Sumatra, Java, Borneo, and the Philippines were interconnected and were separated from Sulawesi by the Makassar Strait.

The systematics of the genus *Paphiopedilum* proposed by Cribb (1997b) are largely consistent with Atwood (1984), except that Cribb placed the *Parvisepalum* group within subgenus *Brachypetalum*. Cribb (1997b) accepted the suggestion of Karasawa (1982) and Karasawa and Saito (1982) to promote the *Parvisepalum* group (e.g., *Parvisepalum delenatii*, *Parvisepalum armeniacum*, *Parvisepalum micranthum*, *Parvisepalum malipoense,* and *Parvisepalum emersonii*) to the subgeneric rank, since the two relatively new species (i.e., *P. malipoense* and *P. emersonii*) found in this group have been described. According to the classification of Cribb (1997b), the genus *Paphiopedilum* comprised of approximately 69 species worldwide. Cribb divided this genus into three subgenera, *Parvisepalum*, *Brachypetalum*, and *Paphiopedilum,* which are mainly based on the morphological characteristics of flower inflorescence, leaf type, floral morphology, and molecular data on ITS sequences (Cox et al., 1997). Recently, several new species and treatment have been described for this genus. The genus *Paphiopedilum* was described as containing approximately 98 species worldwide by the year 2000 (Koopowitz, 2000). In this genus, *Paphiopedilum* was divided into five sections: *Coryopedilum*, *Pardalopetalum*, *Cochlopetalum*, *Paphiopedilum,* and *Barbata*. Subgenera of the genus *Paphiopedilum* distribute in distinct geographic regions (Cribb, 1997b). The subgenera *Parvisepalum* and *Brachypetalum*, as well as the section *Paphiopedilum* of the subgenus *Paphiopedilum*, are found only in mainland Asia. The *Parvisepalum* subgenus is concentrated in southern China and Vietnam, the subgenus *Brachypetalum* is mostly found in Thailand (**Figure 1**). Among the subgenus *Paphiopedilum*, *Paphiopedilum* ranges from India to southern China, Thailand and Indo-China, and the species diversity found in southern China was the most concentrated. The section *Cochlopetalum* is restricted to the islands of Sumatra and Java. The section *Pardalopetalum* is widespread in Southeast Asia, the Malay Archipelago, as far east as Sulawesi, and Luzon in the Philippines. The enormous species diversity of the section *Coryopedilum* locates in Borneo, and this section range from the Philippines to Sulawesi in New Guinea. The section *Barbata* is widespread from eastern Nepal, across to Hong Kong and the Philippines, south to the Malay Archipelago, New Guinea, and the Solomon Islands (Cribb, 1998).

*Paphiopedilum* is a genus of tropical Asiatic origin, and its range extends eastward, reaching the Philippines, Southeast Asia, Borneo, and the Malay Archipelago, crossing Wallace's Line into Sulawesi, the Moluccas, New Guinea, and the Solomon Islands (Cribb, 1998). Tracking back to the geological history of Southeast Asian, the Palawan, Mindoro, Zamboanga, and the adjacent small islands are the older islands of the Southern Philippines. These regions are located on the border of the Eurasian Plate and have been shifting away from the mainland mass by tectonic collision since the early Miocene (~30 Mya) and the shell of the older plate was merged to Borneo until 5~10 Mya (Karig et al., 1986; Stephan et al., 1986; Hall, 1996). In contrast, most of the Philippine islands formed less than 5 Mya (Aurelio et al., 1991; Quebral et al., 1994). In addition, the Sundaland was comprised of the Malay Peninsula, Sumatra, Java, Sulawesi, and Borneo and merged with Bali, the Philippines, and even New Guinea/Australia into Sunda Superland interconnecting by land bridge during the last glacial period (0.01~1.8 Mya) (van Oosterzee, 1997). Since the last glacial period, species migrated forward and backwards between these regions and isolated after the last glacial maximum (LGM), causing the broken of Sunda Superland (Tsai et al., 2015).

The chloroplast primers for the *atp*B-*rbc*L, *trn*L-*trn*F spacer, and *trn*L intron are useful for phylogenetic studies at the intrageneric level. The primers for the *trn*L-*trn*F spacer and *trn*L intron developed by Taberlet et al. (1991) have been applied for inferring phylogenies at the intrageneric level (Goldblatt et al., 2002; Hodkinson et al., 2002; Mogensen, 1996; Van Raamsdonk et al., 2003), and have also been used successfully on Orchidaceae (Tsai et al., 2012). The *atp*B-*rbc*L regions are high length differences due to frequent occurrence of indels and are often used in combination with other primers to provide more information (Yoshinaga et al., 1992; Chiang et al., 1998; Von Konrat et al., 2010). Therefore, this study aims to further elucidate the phylogeny of *Paphiopedilum* through analysis ITS (internal transcribed spacer) sequences and three non-coding plastid DNA sequences (*trn*L intron, *trn*L-F, and *atp*B-*rbc*L spacers). In addition, the biogeography of this genus is clarified based on the phylogenetic tree derived from the molecular evidence.

## Materials and Methods

### Plant Materials

Seventy-eight taxa of *Paphiopedilum* and two outgroups from genus *Phragmipedium* were used in this study (**Table 1**). All leaf materials were taken from living plants in the greenhouse of the Kaohsiung District Agricultural Improvement Station (KDAIS) in Taiwan.

**Table 1:** Names of specimens, geographical distribution, source, and GenBank accession numbers for sequences of the internal transcribed spacer (ITS) of ribosomal DNA (rDNA), the plastid *trn*L intron, the *trn*L-F spacer, and the *atp*B-*rbc*L spacer.

Taxa and systematic classification ^a^	Geographical distribution	Voucher ^b^	GenBank accession no.			
			ITS	*trn*L intron	*trn*L-F spacer	*atp*B-*rbc*L spacer
Genus *Paphiopedilum*						
Subgenus *Parvisepalum*						
*Paphiopedilum armeniacum* S.C. Chen & F.Y. Liu	Southwest China	C. C. Tsai 2021	EF156086	EF156001	EF156171	GQ850803
*Paphiopedilum delenatii* Guill.	Vietnam	C. C. Tsai 2073	EF156096	EF156011	EF156181	GQ850813
*Paphiopedilum emersonii* Koop. & P.J. Cribb	China	C. C. Tsai 2351	EF156099	EF156014	EF156184	GQ850816
* Paphiopedilum hangianum* Perner & Gruss	China	C. C. Tsai 2201	EF156109	EF156024	EF156194	GQ850826
* Paphiopedilum jackii* H.S. Hua	China, Vietnam	C. C. Tsai 2330	EF156118	EF156033	EF156203	GQ850832
* Paphiopedilum malipoense* S.C. Chen & Z.H. Tsi	China, Vietnam	C. C. Tsai 2024	EF156125	EF156040	EF156210	GQ850839
*Paphiopedilum micranthum* T. Tang & F. T. Wang	China, Vietnam	C. C. Tsai 2020	EF156128	EF156043	EF156213	GQ850842
*Paphiopedilum micranthum* var. *eburneum* Fowlie	China	No voucher	EF156127	EF156042	EF156212	GQ850841
*Paphiopedilum vietnamense* Perner & Gruss	Vietnam	C. C. Tsai 2110	EF156158	EF156073	EF156243	GQ850871
Subgenus *Brachypetalum*						
*Paphiopedilum concolor* (Bateman) Pfitzer	China, Burma, Thailand, Laos, Vietnam	C. C. Tsai 2307	EF156093	EF156008	EF156178	GQ850810
*Paphiopedilum godefroyae* (God.-Leb.) Stein	Thailand	C. C. Tsai 2321	EF156107	EF156022	EF156192	GQ850824
*Paphiopedilum godefroyae* var. *leucochilum* (Masters) Hallier	Thailand	C. C. Tsai 2031	EF156106	EF156021	EF156191	GQ850823
*Paphiopedilum niveum* (Rchb.f.) Stein	Southern Thailand, Malay peninsula	C. C. Tsai 2039	EF156130	EF156045	EF156215	GQ850844
**Subgenus** *Paphiopedilum*						
** Section *Coryopedilum***						
*Paphiopedilum adductum* Asher	Philippines	C. C. Tsai 2025	EF156082	EF155997	EF156167	GQ850799
*Paphiopedilum anitum* Golamco	Philippines	C. C. Tsai 2295	EF156083	EF155998	EF156168	GQ850800
*Paphiopedilum gigantifolium* Braem, M.L. Baker & C.O. Baker	Sulawesi	No voucher	EF156103	EF156018	EF156188	GQ850821
*Paphiopedilum kolopakingii* Fowlie	Borneo	C. C. Tsai 2057	EF156121	EF156036	EF156206	GQ850835
*Paphiopedilum ooii* Koopowitz	Borneo	no voucher	EF156138	EF156046	EF156216	GQ850845
*Paphiopedilum philippinense* (Rchb.f.) Stein	Northeast Borneo, Philippines	C. C. Tsai 2007	EF156142	EF156050	EF156220	GQ850848
*Paphiopedilum glanduliferum* (Blume) Stein	New Guinea	C. C. Tsai 2040	EF156104	EF156019	EF156189	GQ850822
*Paphiopedilum randsii* fowlie	Mindanao, Philippines	C. C. Tsai 2297	EF156132	EF156053	EF156223	GQ850851
*Paphiopedilum rothschildianum* (Rchb.f.) Stein	Borneo	C. C. Tsai 2249	EF156135	EF156056	EF156226	GQ850853
*Paphiopedilum sanderianum* (Rchb.f.) Stein	Borneo	C. C. Tsai 2309	EF156136	EF156057	EF156227	GQ850854
*Paphiopedilum stonei* (Hook.) Stein	Borneo	C. C. Tsai 2310	EF156146	EF156061	EF156231	GQ850858
*Paphiopedilum supardii* Braem & Loeb	Borneo	C. C. Tsai 2189	GQ505309	GQ505312	GQ505315	GQ850860
*Paphiopedilum wilhelminae* L.O. Williams	New Guinea	C. C. Tsai 2205	GQ505310	GQ505313	GQ505316	GQ850875
** Section** *Pardalopetalum*						
*Paphiopedilum dianthum* T. Tang & F.T. Wang	China	C. C. Tsai 2085	EF156097	EF156012	EF156182	GQ850814
*Paphiopedilum haynaldianum* (Rchb.f.) Stein	Philippines	No voucher	EF156110	EF156025	EF156195	GQ850827
*Paphiopedilum lowii* (Lindl.) Stein	Peninsular Malaysia, Sumatra, Borneo, Sulawesi	C. C. Tsai 2285	EF156124	EF156039	EF156209	GQ850838
*Paphiopedilum parishii* (Rchb.f.) Stein	Southwest China, Burma, Thailand	C. C. Tsai 2276	EF156140	EF156048	EF156218	GQ850847
*Paphiopedilum richardianum* Asher & Beaman	Sulawesi	C. C. Tsai 2068	EF156133	EF156054	EF156224	GQ850852
** Section** *Cochlopetalum*						
*Paphiopedilum victoria-regina* (Sander) M.W. Wood	Sumatra	C. C. Tsai 2045	EF156157	EF156072	EF156242	GQ850870
*Paphiopedilum liemianum* (Fowlie) Karas. & Saito	Northern Sumatra	C. C. Tsai 2133	EF156123	EF156038	EF156208	GQ850837
*Paphiopedilum moquetteanum* (J.J. Smith) Fowlie	Southwest Java	C. C. Tsai 2314	EF156129	EF156044	EF156214	GQ850843
*Paphiopedilum primulinum* M.W. Wood & P. Taylor	Northern Sumatra	C. C. Tsai 2359	EF156143	EF156051	EF156221	GQ850849
*Paphiopedilum victoria-mariae* (Rolfe) Rolfe	Sumatra	No voucher	EF156156	EF156071	EF156241	GQ850869
** Section** *Paphiopedilum*						
*Paphiopedilum barbigerum* Tang & Wang	China, northern Vietnam	C. C. Tsai 2023	EF156088	EF156003	EF156173	GQ850805
*Paphiopedilum charlesworthii* (Rolfe) Pfitzer	Burma, northern Thailand, southwest China	C. C. Tsai 2192	EF156091	EF156006	EF156176	GQ850808
*Paphiopedilum druryi* (Bedd.) Stein	Southern India	C. C. Tsai 2093	EF156098	EF156013	EF156183	GQ850815
*Paphiopedilum exul* (Ridl.) Rolfe	Peninsular Thailand	C. C. Tsai 2083	EF156101	EF156016	EF156186	GQ850818
*Paphiopedilum esquirolei* Schltr.	China, India, Bhutan (Southeast Asia)	C. C. Tsai 2335	EF156100	EF156015	EF156185	GQ850817
*Paphiopedilum fairrieanum* (Lindl.) Stein	India, Bhutan	C. C. Tsai 2079	EF156102	EF156017	EF156187	GQ850819
*Paphiopedilum gratrixianum* (Masters) Guillaumin	Laos, Vietnam	C. C. Tsai 2155	EF156108	EF156023	EF156193	GQ850825
*Paphiopedilum helenae* Aver.	Northern Vietnam	C. C. Tsai 2053	EF156111	EF156026	EF156196	GQ850828
*Paphiopedilum henryanum* Braem	China, northern Vietnam	C. C. Tsai 2277	EF156112	EF156027	EF156197	GQ850829
*Paphiopedilum herrmannii* Fuchs & Reisinger	Northeast India	C. C. Tsai 2109	EF156113	EF156028	EF156198	GQ850880
*Paphiopedilum hirsutissimum* (Lindl. ex Hook.) Stein	China, India, Bhutan (Southeast Asia)	C. C. Tsai 2240	EF156114	EF156029	EF156199	GQ850830
*Paphiopedilum spicerianum* (Rchb.f.) Pfitzer	Northeast India	C. C. Tsai 2229	EF156145	EF156060	EF156230	GQ850857
*Paphiopedilum tigrinum* Koop. & N. Haseg.	China	C. C. Tsai 2218	EF156149	EF156064	EF156234	GQ850862
*Paphiopedilum tranlienianum* Gruss & Perner	Unknown	C. C. Tsai 2042	EF156151	EF156066	EF156236	GQ850864
*Paphiopedilum villosum* (Lindl.) Stein	India, Burma, Thailand (Southeast Asia)	C. C. Tsai 2216	EF156159	EF156074	EF156244	GQ850872
** Section** *Barbata*						
*Paphiopedilum acmodontum* Schoser ex M.W. Wood	Philippines	C. C. Tsai 2094	EF156081	EF155996	EF156166	GQ850879
*Paphiopedilum appletonianum* (Gower) Rolfe	China, Thailand, Cambodia, Laos, Vietnam (Southeast Asia)	C. C. Tsai 2153	EF156084	EF155999	EF156169	GQ850801
*Paphiopedilum argus* (Rchb.f.) Stein	Philippines	C. C. Tsai 2282	EF156085	EF156000	EF156170	GQ850802
*Paphiopedilum braemii* Mohr	Northern Sumatra, Indonesia	C. C. Tsai 2151	EF156089	EF156004	EF156174	GQ850806
*Paphiopedilum barbatum* (Lindl.) Pfitzer	Southern Thailand, peninsular Malaysia, Sumatra	C. C. Tsai 2227	EF156087	EF156002	EF156172	GQ850804
*Paphiopedilum callosum* (Rchb.f.) Stein	Thailand, Cambodia, Laos, Vietnam (south-east Asia)	C. C. Tsai 2267	EF156090	EF156005	EF156175	GQ850807
*Paphiopedilum ciliolare* (Rchb.f.) Stein	Philippines	C. C. Tsai 2078	EF156092	EF156007	EF156177	GQ850809
*Paphiopedilum curtisii* (Rchb. f.) Stein	Sumatra	C. C. Tsai 2107	EF156094	EF156009	EF156179	GQ850811
*Paphiopedilum dayanum* (Lindl.) Stein	Borneo	C. C. Tsai 2280	EF156095	EF156010	EF156180	GQ850812
*Paphiopedilum fowliei* Birk	Philippines	No voucher	GQ505311	GQ505314	GQ505317	GQ850820
*Paphiopedilum hookerae* (Rchb.f.) Stein	Borneo	C. C. Tsai 2089	EF156116	EF156031	EF156201	GQ850831
*Paphiopedilum volonteanum* (Sander) Stein	Borneo	No voucher	EF156115	EF156030	EF156200	GQ850873
*Paphiopedilum javanicum* (Reinw. ex Lindl.) Pfitzer	Borneo, southeast Sumatra, Java	C. C. Tsai 2326	EF156120	EF156035	EF156205	GQ850834
*Paphiopedilum javanicum* var. *virens* (Rchb. f.) Stein	North Borneo	No voucher	EF156119	EF156034	EF156204	GQ850833
*Paphiopedilum lawrenceanum* (Rchb.f.) Stein	Borneo	C. C. Tsai 2013	EF156122	EF156037	EF156207	GQ850836
*Paphiopedilum mastersianum* (Rchb.f.) Stein	Moluccas	C. C. Tsai 2341	EF156126	EF156041	EF156211	GQ850840
*Paphiopedilum papuanum* (Ridl.) Ridl.	New Guinea	No voucher	EF156139	EF156047	EF156217	GQ850846
*Paphiopedilum purpuratum* (Lindl.) Stein	China, Vietnam	C. C. Tsai 2049	EF156131	EF156052	EF156222	GQ850850
*Paphiopedilum sangii* Braem	Sulawesi	C. C. Tsai 2088	EF156137	EF156058	EF156228	GQ850855
*Paphiopedilum schoseri* Braem	Moluccas	No voucher	EF156144	EF156059	EF156229	GQ850856
*Paphiopedilum sukhakulii* Schoser & Senghas	Northern Thailand	C. C. Tsai 2226	EF156147	EF156062	EF156232	GQ850859
*Paphiopedilum superbiens* (Rchb.f.) Stein	Sumatra	C. C. Tsai 2082	EF156148	EF156063	EF156233	GQ850861
*Paphiopedilum tonsum* (Rchb.f.) Stein	Northern Sumatra, Indonesia	C. C. Tsai 2087	EF156150	EF156065	EF156235	GQ850863
*Paphiopedilum urbanianum* Fowlie	Philippines	C. C. Tsai 2161	EF156152	EF156067	EF156237	GQ850865
*Paphiopedilum veniferum* Koop. & Haseg	Unknown	C. C. Tsai 2253	EF156153	EF156068	EF156238	GQ850866
*Paphiopedilum venustum* (Wall. ex Sims) Pfitzer ex Stein	Bhutan, India, Nepal	C. C. Tsai 2032	EF156154	EF156069	EF156239	GQ850867
*Paphiopedilum wardii* Summerh	Burma, southwest China	C. C. Tsai 2139	EF156161	EF156076	EF156246	GQ850874
** Genus** *Phragmipedium*						
*Phragmipedium pearcei* Garay	Ecuador, Peru	C. C. Tsai 2009	EF156163	EF156078	EF156248	GQ850877
*Phragmipedium longifolium* Rchb. f. & Warsc	Costa Rica, Panama, Colombia, Ecuador	C. C. Tsai 2043	EF156165	EF156080	EF156250	GQ850876

a The systematics of Phalaenopsis are based on Christenson (2001).

b Voucher specimens were deposited at the herbarium of the National Museum of Natural Science, Taiwan (TNM).

### DNA Extraction, PCR Amplification, and Sequencing

Total DNA was extracted from fresh etiolated leaves by using the cetyltrimethylammonium bromide (CTAB) method (Doyle and Doyle, 1987). Approximate DNA yields were determined by using the spectrophotometer (model U-2001, Hitachi).

The PCR reaction was used to amplify nuclear ribosomal ITS sequence and chloroplast (cp) DNA fragments *trn*L intron and the *trn*L-*trn*F spacer, *atp*B-*rbc*L spacer. ITS primers were designed from conserved regions of the 3' end of the 18S rRNA gene and the 5'end of the 26S rRNA gene using sequences from different species present in GenBank. Universal primers for *trn*L intron and the *trn*L-*trn*F spacer were referenced from Taberlet et al. (1991). Primer sequences for amplifying of the *atp*B-*rbc*L spacer were designed from the conserved regions of the 3' end of the *atp*B gene and the 5'end of the *rbc*L gene of chloroplast DNA using sequences of different species obtained from GenBank. Detailed amplification conditions and primer sequences are given in **Supplementary Table S1**. All PCR products were separated by agarose gel electrophoresis (1.0%, w/v in TBE) and were recovered using glassmilk (BIO 101, California).

PCR products were directly sequenced using the dideoxy chain-termination method on an ABI377 automated sequencer with the Ready Reaction Kit (PE Biosystems, California) of the BigDye™ Terminator Cycle Sequencing. The PCR reaction primer sequences were used as sequencing primers. Each sample was sequenced two or three times to confirm the sequences. Reactions were performed as recommended by the product manufacturers.

### Sequence Alignment and Phylogenetic Reconstruction

The sequence alignment was determined using the ClustalW multiple alignment program in BioEdit (Hall, 1999), and four regions were combined for the following analysis. The alignment was checked, and apparent alignment errors were corrected by hand. Indels (insertion/deletions) were treated as missing data. For phylogenetic reconstruction, two *Phragmipedium* taxa treating as outgroups were sequenced to resolve whether all in-group taxa formed a monophyletic lineage. The best-fitting substitution model was selected (**Supplementary Table S2**) by a model test using MEGA 6.0 (Tamura et al., 2013). Tamura 3-parameter model (T92) using a discrete Gamma distribution (+G) was selected for following neighbor-joining (NJ) phylogenetic reconstruction. The general time reversible (GTR) using a discrete gamma distribution (+G) and considering the proportion of invariable sites (+I) were chosen for following divergence time estimation using the Yule model methods in BEAST 1.8.0 (Drummond and Rambaut, 2007; Drummond et al., 2012). The phylogenetic tree for the combined multiple sequence datasets used equally weighted characters. Moreover, because the sequence data of the four genera (*Mexipedium, Selenipedium*, *Cypripedium*, and *Goodyera*) in NCBI is limited, only two sets of fragment data (ITS: *Mexipedium xerophyticum*-MK161260.1; *Selenipedium aequinoctiale*-JF825977.1; *Cypripedium_macranthos*-KT338684.1; Goodyera_procera-MK451741.1and *trn*L-*trn*F spacer: *Mexipedium xerophyticum*-FR851215.1; *Selenipedium aequinoctiale*-JF825975.1; *Cypripedium_macranthos*-JF797026.1; Goodyera_procera-MK451782.1) are used as an additional analysis and compared with the data using only genus *Phragmipedium* as outgroup. The results of six outgroups are showed in **Supplementary Data**. Genetic relationships were determined using NJ in the MEGA 6.0 (Tamura et al., 2013), maximum parsimony (MP) in PHYLIP 3.68 (Felsenstein, 2004), and maximum likelihood (ML) in MEGA 6.0 (Tamura et al., 2013). Bootstrapping (1,000 replicates) was carried out to estimate the support for NJ, MP, and ML topologies (Felsenstein, 1985; Hillis and Bull, 1993). The strict consensus parsimonious tree was then constructed by using the MEGA 6.0 (Tamura et al., 2013).

### Divergence Time Estimation

The combined chloroplast DNA (cpDNA) dataset was used to estimate the divergence times using the Bayesian Yule model methods (BEAST version 1.7.5). The characteristic of uniparental inheritance in cpDNA prevents the interference of recombination introgression on phylogenetic reconstruction (Drummond et al., 2012). The general-time reversible (GTR) model with estimates of invariant sites (+I) and gamma-distributed among site rate variation (+G) in all matrices without partitions model was determined by the nucleotide substitution model test, conducted in MEGA 6.0 (Tamura et al., 2013).

To estimate the divergence time, two strategies, the strict and relaxed clock models, were adopted. For the relaxed clock, the calibration point at the most recent common ancestor (MRCA) of *Paphiopedilum* and *Phragmipedium* for 22 Ma (Gustafsson et al., 2010) were used to calculate the divergence times of each node. However, since there is no suitable fossil record to correct the calibration of divergence times for the ingroup, we recalculate the divergence time by strict clock model for consistency. For the strict clock, the mean substitution rate of 1.82 × 10^−9^ subs/site/year with the lower and upper limits 1.11 × 10^−9^ subs/site/year and 2.53 × 10^−9^ subs/site/year, respectively, were used for the cpDNA spacers in *Phalaenopsis amabilis* complex (Tsai et al., 2015).

We conducted four independent runs of a Yule prior and four Markov Chain Monte Carlo (MCMC) chains with a different starting seed. The first 10% simulations were discarded (burn-in) in a total of 10^8^ generations. For thinning, one tree was reserved every 10,000 trees, and finally, 10,000 trees were left to calculate the posterior probability of each node. The effective sample size (ESS) > 200 was used as a criterion to check whether the sampling (simulations) is proper and is reaching a stationary distribution by Tracer v1.6 (Rambaut et al., 2018). Four independent-runs results, including the log file and tree file, were combined with the assistance of LogCombiner 1.6.1 (Drummond and Rambaut, 2007). TreeAnnotator 1.6.1 (Drummond and Rambaut, 2007) was used to summarize a consensus tree with a criterion of the maximum clade reliability using the mean heights option. The final consensus tree was drawn by FigTree 1.3.1 (Rambaut, 2009).

### Biogeographic Inference Using Reconstruct Ancestral State in Phylogenies

The Statistical dispersal–vicariance analysis was used to assess the biogeographic patterns of *Paphiopedilum* species [Statistical Dispersal-Vicariance Analysis (S-DIVA), (Yu et al., 2010)]. Bayes–Lagrange Statistical dispersal–extinction–cladogenesis (S-DEC) model (Ree and Smith, 2008) was performed in Reconstruct Ancestral State in Phylogenies (RASP) 3.2 (Yu et al., 2015) to distinguish the events of vicariance, dispersal, and extinction. Five geographic areas were determined mainly according to Myers et al. (2000) with a little modification to illuminate the vicariance, dispersal and extinction events of *Paphiopedilum* species. The hotspot areas in South-Central China and Indo-Burma with the Malay Peninsula were combined as area A, consisting of China, Nepal, India, Bhutan, Burma, Thailand, Malaysia, Cambodia, Vietnam, and Laos. The hotspot “Sundaland” including Borneo, Java, and Sumatra were set as the area B. We move the Malay Peninsula from the area “Sundaland” to area A due to the integrity of the current landmass. The hotspots “Wallacea” (include Sulawesi and Moluccas) and “Philippines” were set as area C and area E. Respectively, islands of New Guinea eastern from the Wallacea are defined as area D. Species of outgroup were all defined as the area I. These two outgroup species are distributed in Ecuador, Peru, Costa Rica, Panama, and Colombia. The real distribution of outgroups is too far from the areas of the species in this study. Therefore, the ranges of outgroups are assigned to a new area in which none of the ingroup species occurs (Yu et al., 2014). The ML tree topologies were used in S-DIVA analysis.

## Results

### Sequence Alignment and Characteristics

The lengths of the ITS sequences obtained from the *Paphiopedilum* and outgroup samples were similar to those reported for a broad example of angiosperms (Baldwin, 1992; Baldwin et al., 1995). The alignment length of the ITS sequence is 735 nucleotides, of which 343 were identified as variable sites with 235 potentially parsimony informative sites. The average genetic distance between the 78 *Paphiopedilum* samples was 0.039 in ITS, and the average genetic distance between the 78 *Paphiopedilum* species was 0.01 in cpDNA. The alignment of combined plastid DNA fragments contained a total of 2,409 characters, of which 872 were identified as variable sites with 588 potentially parsimony informative sites. Since the sequences of three samples of every species are the same within species, only one sequence per species was used for the analyses and deposited in NCBI GenBank. The accession numbers of the nuclear ribosomal ITS sequences and the three fragments of plastid DNA from the 78 *Paphiopedilum* taxa and the two outgroup samples (from the genus *Phragmipedium*) are shown in **Table 1**.

### Phylogeny Reconstruction

**Figure 2:**
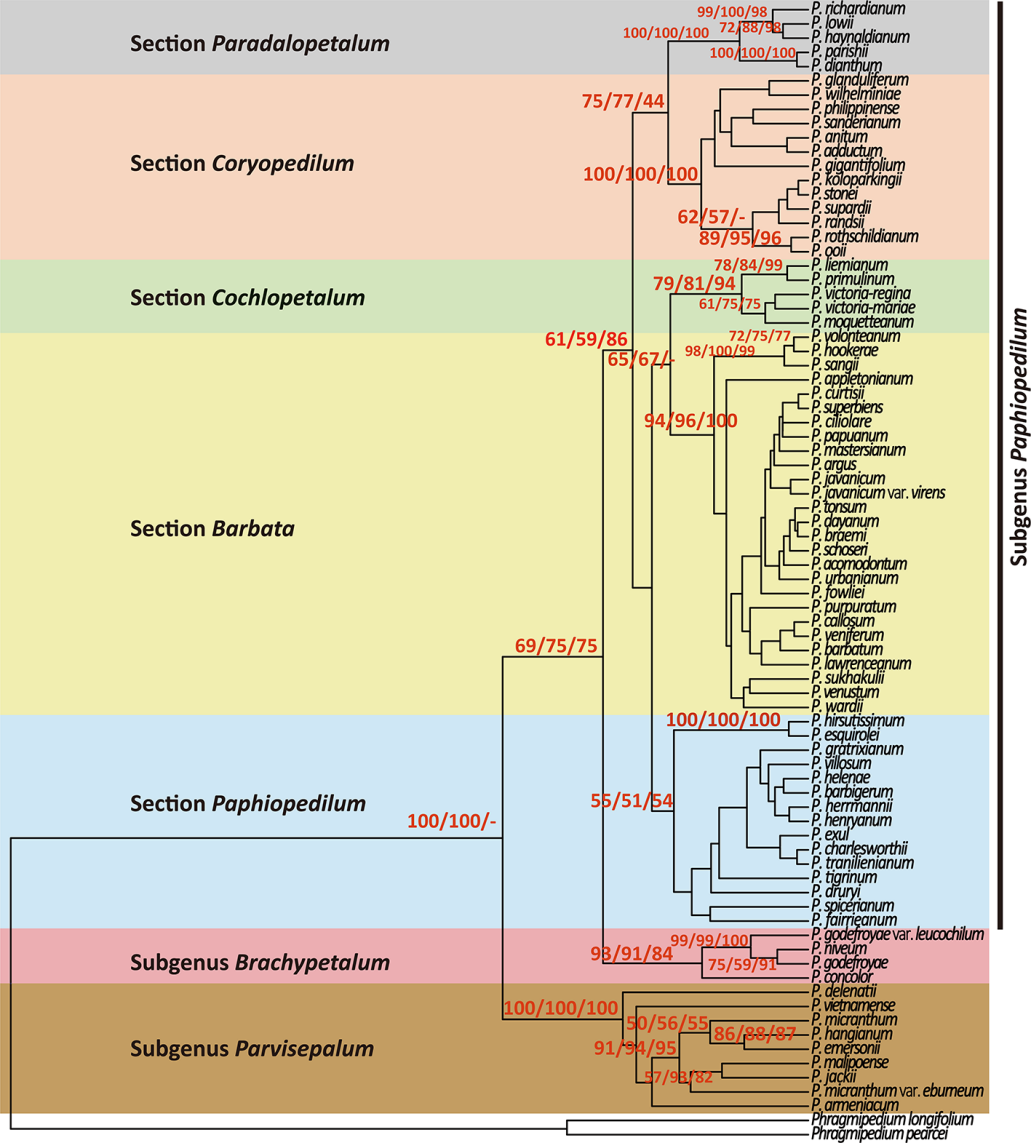
Phylogenetic relationships using neighbor-joining (NJ), maximum parsimony (MP), and maximum likelihood (ML) resulting from analysis of the combined data matrix (nuclear ribosomal ITS, plastid *trn*L intron, *trn*L-F spacer, and *atp*B-*rbc*L spacer) from 78 *Paphiopedilum* and 2 outgroup species. Only the strict consensus of all most parsimonious trees (MP trees) are showed in this figure, and the bootstrap values > 50% are shown on each branch for NJ/MP/ML between major lineage.

Both NJ and MP trees revealed a monophyletic relationship of 78 *Paphiopedilum* taxa with high bootstrap supports (**Figure 2**). Moreover, the use of six outgroups for phylogeny reconstruction showed similar bootstrap supports (**Supplementary Figure S1**). In the *Paphiopedilum* monophyletic clade, three subgenera *Parvisepalum*, *Brachypetalum*, and *Paphiopedilum* formed independent monophyletic clades with 100/100/100, 98/91/84, and 61/59/86% bootstrap supporting values in NJ/MP/ML trees, in which subgenus *Parvisepalum* was diverged firstly from other lineages (**Figure 2**, **Supplementary Figure S1**). In subgenus *Paphiopedilum*, sections *Barbata*, *Cochlopetalum, Pardalopetalum,* and *Coryopedilum* are monophyletic with 94/96/100, 79/81/94, 100/100/100, and 100/100/100% bootstrap supporting values in NJ, MP, and ML trees. Additionally, the use of six outgroups for phylogeny reconstruction showed similar patterns (**Supplementary Figure S1**). However, section *Paphiopedilum* showed a low support [56/51/54% bootstrap values (**Supplementary Figure S2**); 59% bootstrap values (**Supplementary Figure S1**)] for the monophyly.

**Figure 3:**
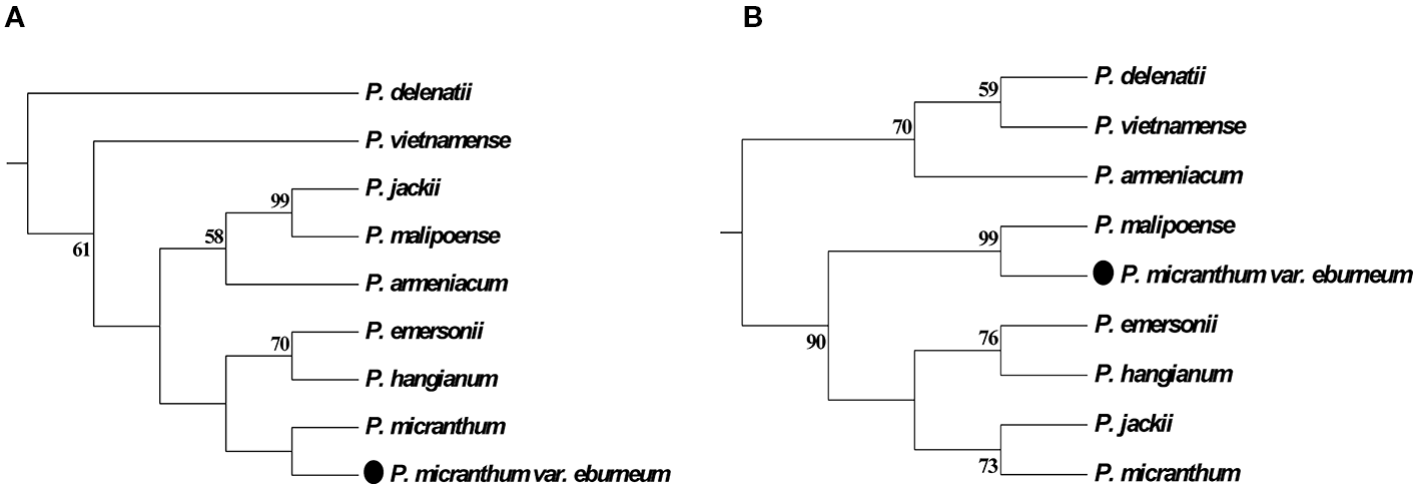
Parsimonious phylogenetic tree of the *Paphiopedilum* subgenus *Parvisepalum* derived from ITS sequences of nuclear ribosomal DNA (nrDNA) **(A)** and plastid DNA **(B)**. The solid circle (․) represents a putative natural hybrid (based on molecular evidence).

In addition, the molecular data demonstrates that a newly described variety, *P. micranthum* var. *eburneum*, is closely related to *P. malipoense* based on the plastid DNA within subgenus *Parvisepalum*, which is inconsistent with the inference by nuclear ITS and combined data. In ITS tree, *P. micranthum* var. *eburneum* is sister with *P. micranthum* var. *micranthum* (**Figures 2** and **3**). Therefore, we infer a hybridization event between the ancestor of *P. micranthum* and *P. malipoense* that lead to a plastid capture in *P. micranthum* var. *eburneum*.

### Divergence Time Estimates

**Figure 4:**
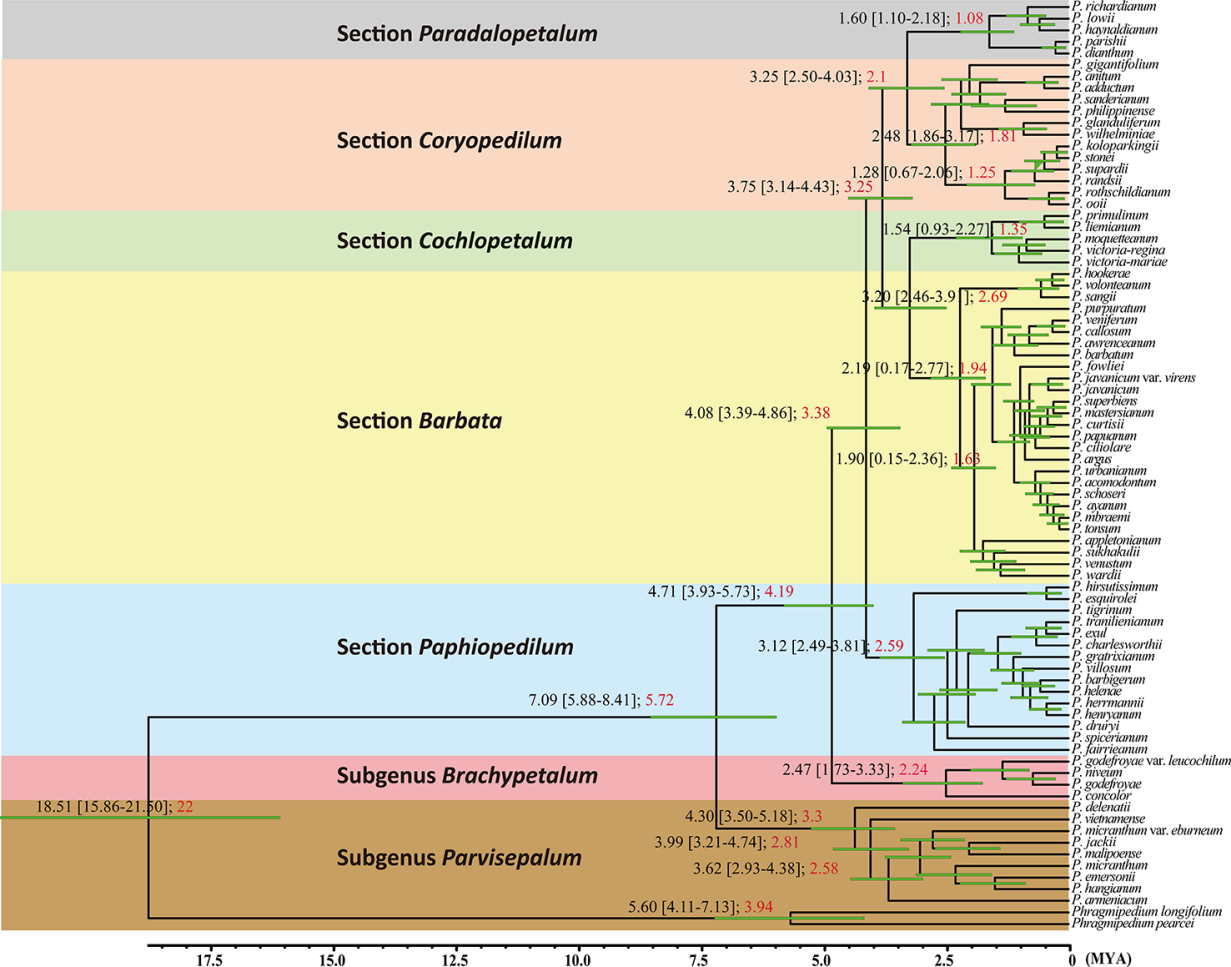
Results of calescence time estimations performed with BEAST 1.8.0 for the from 78 *Paphiopedilum* taxa based on the combined data matrix (nuclear ribosomal ITS, plastid *trn*L intron, *trn*L-F spacer, and *atp*B-*rbc*L spacer). The black numbers in each node are using the mean rate of 1.82 × 10−9 subs/site/year, with the lower and upper limits 1.11 × 10−9 subs/site/year and 2.53 × 10−9 subs/site/year (Tsai et al., 2015). The red numbers in each node are using the Gustafsson et al. (2010) fossil calibration time data to calibrate the divergence time.

The coalescence time of the genus *Paphiopedilum* was estimated to be 7.09 Mya with 95% confidence intervals (95% CI) of 5.88–8.41 Mya (**Figure 4**) according to the substitution rate referenced from Tsai et al. (2015). If calibrating by relaxed clock referring to Gustafsson et al. (2010), the estimated coalescence time of the genus *Paphiopedilum* was 5.72 Mya (**Figure 4**). In the genus *Paphiopedilum*, the coalescence times estimated by strict clock were 4.30 Mya (95% CI: 3.50–5.18 Mya), 2.47 Mya (95% CI: 1.73–3.33 Mya), and 4.08 Mya (95% CI: 3.39–4.86 Mya) for subgenera *Parvisepalum*, *Brachypetalum*, and *Paphiopedilum*, respectively (**Figure 4**). After re-calibrating by a relaxed clock, the coalescence time was estimated to be 3.3 Mya, 2.24, and 3.38 Mya Mya for subgenera *Parvisepalum*, *Brachypetalum*, and *Paphiopedilum*, respectively (**Figure 4**). In addition, the strict clock suggested that the coalescence times were tracked back to 2.19 Mya (95% CI: 0.17–2.77 Mya), 1.54 Mya (95% CI: 0.93–2.27 Mya), 3.12 Mya (95% CI: 2.49–3.81 Mya), 2.48 (95% CI: 1.86–3.17 Mya), and 1.60 Mya (95% CI: 1.10–2.18 Mya) for clades of subgenera *Barbata*, *Cochlopetalum*, *Paphiopedilum*, *Coryopedilum,* and *Pardalopetalum* of subgenus *Paphiopedilum*, respectively. By relaxed clock, the coalescence time was estimated to be 1.94, 1.3, 2.59, 1.81, and 1.08 Mya for clades of subgenera *Barbata*, *Cochlopetalum*, *Paphiopedilum*, *Coryopedilum,* and *Pardalopetalum* of subgenus *Paphiopedilum*, respectively. Additionally, the use of six outgroups for divergence time estimation also showed similar supports (**Supplementary Figure S2**). In short, the estimates of the coalescence times by strict and relaxed clocks are similar, but the time calculated is slightly shorter by relaxed clocks. Regardless, the coalescence time of the genus *Paphiopedilum* will not be earlier than Upper Miocene.

### Demographic History and Historical Biogeography Inference

**Figure 5:**
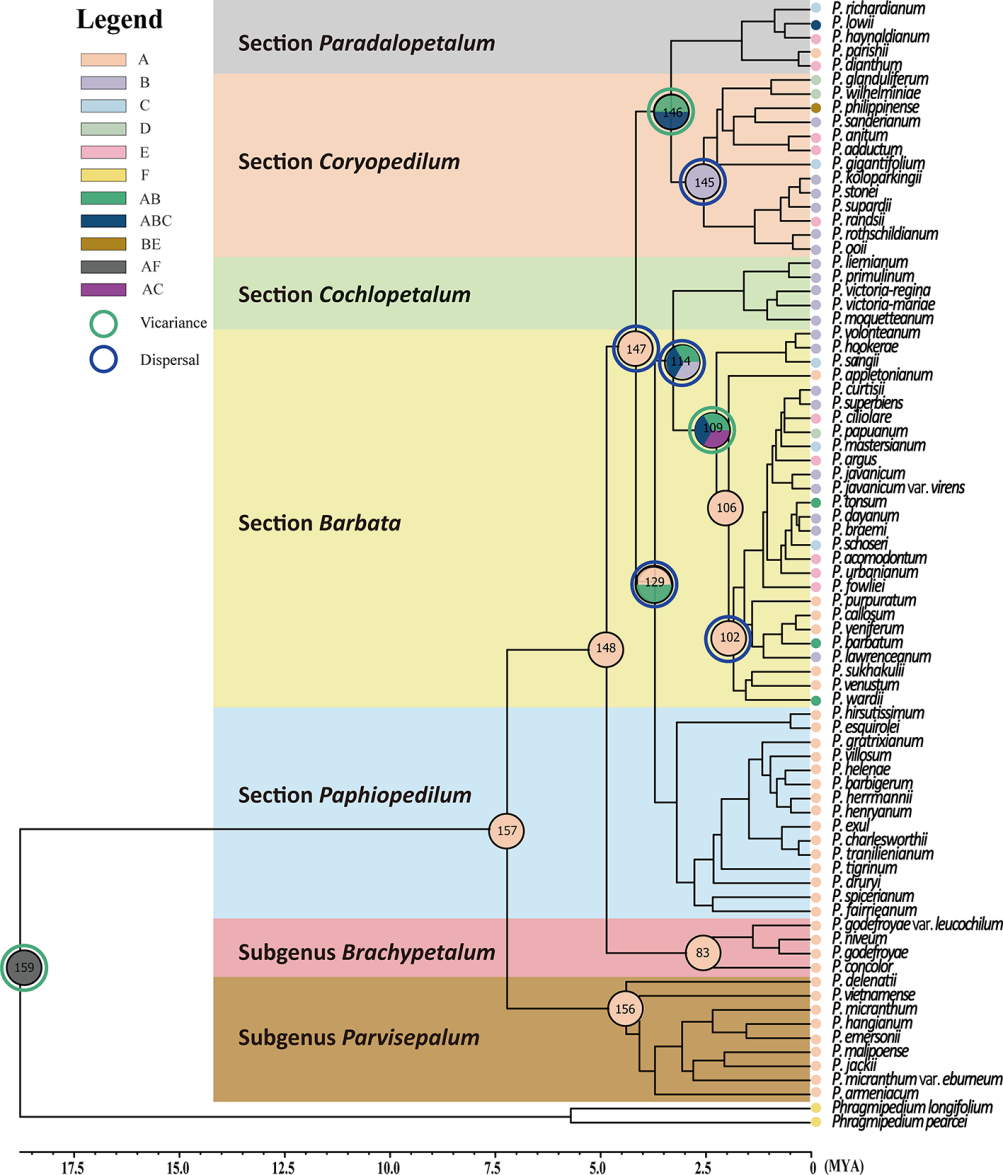
Ancestral distributions reconstructed by the Statistical dispersal–vicariance analysis [S-DIVA, (Yu et al., 2010)] and Bayes–Lagrange Statistical dispersal–extinction–cladogenesis (S-DEC) model (Ree and Smith, 2008) performed in Reconstruct Ancestral State in Phylogenies (RASP) 3.2 (Yu et al., 2015). Phylogenetic relationships of the 78 *Paphiopedilum* species, plus the two outgroups *Phragmipedium pearcei* and *Ph. longifolium*, obtained from sequence judgments of the combined sequence and generated by BEAST. The distribution areas of extant the 78 *Paphiopedilum* species are marked in capitals A–E and I [(A) China, Nepal, India, Bhutan, Burma, Thailand, Malaysia, Cambodia, Vietnam, and Laos; (B) Sumatra, Borneo and Java; (C) Sulawesi and Moluccas; (D) New Guinea; (E) Philippines; and (I) outgroup], respectively. The green and blue circles indicate the vicariance and dispersal events obtained from the RASP analysis, respectively.

Complicated evolutionary processes of continuous and episodic dispersal, vicariance, and extinctions determined the current geographic distribution of genus *Paphiopedilum*. Since the most probable ancestral areas located on continental Asia (area A in **Figure 5**), dispersal events seem to determine the extant distributions of subgenera largely. The results supported vicariance events on nodes 159, 146, and 109 shown in **Table 2** and **Figure 5**, and on nodes 163, and 132 (**Supplementary Table S3** and **Figure S3**). The node 147 revealed dispersal events among section *Coryopedilum/Pardalopetalum* of *Paphiopedilum* and other sections of *Paphiopedilum* causing by migration route from area A (China, Nepal, India, Bhutan, Burma, Thailand, Malaysia, Cambodia, Vietnam, and Laos) to B area (Sumatra, Borneo, and Java). Meanwhile, the nodes 145, 129, 114, and 102 also revealed dispersal events from north to south, according to Sundaland and Sunda Super Islands.

**Table 2:** The ancestral areas and dispersal–vicariance analysis inferred through Reconstruct Ancestral State in Phylogenies (RASP). Ancestral areas for the node and the number of dispersal (Dis), vicariance (Vic), and extinction (Ext) events are shown.

Node	Ancestral areas	RASP ROUTE	Dis	Vic	Ext	Prob
159	[AF]	AF- > A|F	0	1	0	1.00
157	[A]	A- > A^A- > A|A	0	0	0	1.00
156	[A]	A- > A^A- > A|A	0	0	0	1.00
148	[A]	A- > A^A- > A|A	0	0	0	1.00
147	[A]	A- > A^A^B- > ABC^A^B- > AB|ABC	3	0	0	0.99
146	[ABC|AB]	ABC- > AC|B	0	1	0	1.00
145	[B]	B- > B^B- > BDE^B- > BDE|B	2	0	0	0.91
129	[AB|A]	AB- > AB^A- > ABC^A- > A|ABC	2	0	0	0.74
114	[ABC|AB|B]	ABC- > ABC^B- > B|ABC	1	0	0	0.60
109	[ABC|AC|AB]	ABC- > BC|A	0	1	0	1.00
106	[A]	A- > A^A- > A|A	0	0	0	0.89
102	[A]	A- > A^A- > ABE^A- > A|ABE	2	0	0	0.99
83	[A]	A- > A^A- > A|A	0	0	0	1.00

Furthermore, the use of six outgroups for dynamic historical inference showed similar patterns (**Supplementary Table S3** and **Figure S3**). Only the nodes 146 and 109 were detected vicariance event causing by the geological separation between Indochina and Sumatra/Borneo/Java. In addition, in subgenus *Paphiopedilum*, 2 vicariance and 10 dispersal events were detected, which suggesting a significant dispersal process affected biogeographical patterns in shaping the current distribution in the subgenus *Paphiopedilum*. Areas A and B might be the two possible ancestral areas and likely shaped by several complicated dispersal events in the subgenus *Paphiopedilum*.

## Discussion

### Systematics Revision of Genus *Paphiopedilum*

In general, our phylogenetic inference is mostly congruent with that of Cox et al. (1997), Cribb (1997b), and Guo et al. (2015). In the genus *Paphiopedilum*, tessellated leaves, single flowers with broad elliptic to subcircular petals, and a sizeable thin-textured lip characterize subgenus *Parvisepalum* in southwest China and Vietnam (Cribb, 1998). Within this subgenus, the phylogenetic topography and the divergence time of at least 4.30 Mya rejected the previous hypothesis of the sister-species relationship between *P. armeniacum* and *P. delenatii* (Cribb, 1983) (**Figure 4**). The geographical distribution of these two species is also separated (Yunnan, China for *P. armeniacum,* and Vietnam for *P. delenatii*) (Cribb, 1998).

Furthermore, a newly described variety, *P. micranthum* var. *eburneum*, is phylogenetically close to *P. malipoense* in maternal-inherited plastid DNA but close to *P. micranthum* in biparental-inherited nuclear ITS sequences (**Figure 5**), suggesting that *P. micranthum* var. *eburneum* is a natural hybrid between the maternal parent *P. malipoense* and the paternal parent *P. micranthum* and experienced the event of chloroplast capture. The overlap of the geographical distribution of these three taxa also supports this hypothesis (Cribb, 1998). In addition, ITS sequences are usually concertedly evolved *via* unequal crossing-over (Schlotterer and Tautz, 1994) and biased gene conversion (Hillis et al., 1991), which results in sequence homogeneity between paralogs (Maynard, 1989).

The monophyly of subgenus *Brachypetalum* inferred in this study is congruent with the inference by Cox et al. (1997). The subgenus *Brachypetalum* is geographically confined to Southeast Asia (Cribb, 1998). Albeit overlapping distribution with subgenus *Parvisepalum* (Cribb, 1998), subgenus *Brachypetalum* is phylogenetically separated, consistent with the distinguishable leaf anatomy between these two subgenera (Cribb, 1998). Both molecular and morphological evidences support the independent taxonomic treatment between subgenera *Brachypetalum* and *Parvisepalum* (Karasawa, 1982; Karasawa and Saito, 1982; Cribb, 1997b), but object with Atwood's (1984) opinion of taking the subgenus *Parvisepalum* as a synonym of *Brachypetalum*.

The monophyletic subgenus *Paphiopedilum* can be morphologically and phylogenetically subdivided into five sections: *Coryopedilum*, *Pardalopetalum*, *Cochlopetalum*, *Paphiopedilum*, and *Barbata* (Cox et al., 1997; Cribb, 1997b). Section *Coryopedilum* is characterized by its plain green, strap-like leaves, and multi-flowered inflorescences, which flowers are blooming simultaneously (Cribb, 1998). This section distributes throughout Borneo, Sulawesi, New Guinea, and the Philippines (Cribb, 1998). Except placing *Paphiopedilum parishii* and *Paphiopedilum dianthum* into section *Pardalopetalum* from section *Coryopedilum*, Cribb (1997b) agreed with Atwood (1984) that section *Pardalopetalum* is independent from section *Coryopedilum* taxonomically, according to the ITS analysis (Cox et al., 1997) and similar green strap-like leaves and staminodes (Cribb, 1997b), which is consistent with our phylogenetic inference. However, the only character that separates sections of *Pardalopetalum* and *Coryopedilum* is the morphology of staminode. Whether this single character is sufficient to characterize them as separating sections should be re-evaluated with more evidence.

Unlike the simultaneous bloom of section *Coryopedilum*, section *Cochlopetalum* flower in succession, and their flowers bear elliptic bracts, linear, spirally twisted, spreading, ciliate petals, and a pot-shaped spotted lip (Cribb, 1998). Section *Cochlopetalum* distributes in Sumatra and Java only (Cribb, 1998). The extensive section *Barbata* is the sister of section *Cochlopetalum*, also characterized by a solitary flower with a lip and prominent incurved side-lobes, but the leaf tessellated (Cribb, 1998). The morphological dissimilarity and reciprocally monophyletic relationship indicate that, despite recently diverged, these two sections should be independent taxonomically.

### Biogeography and Evolutionary Trends

The clade of genus *Paphiopedilum* is coalesced to 7.09 or 5.72 Mya, similar to the estimate of 7.62 Mya by Guo et al. (2015). The flower morphology of subgenus *Parvisepalum* is intermediate between other subgenera of *Paphiopedilum* and *Cypripedium* (Chen and Tsi, 1984), which could be explained by the earlier divergence of subgenus *Parvisepalum* in genus *Paphiopedilum* (Guo et al., 2012). Presently, the genus *Cypripedium* is distributed throughout worldwide temperate zones (Cox et al., 1997), with China as a center for species diversity (Cribb, 1997a). Therefore, genera *Paphiopedilum* and *Cypripedium* have most likely diverged in mainland Asia (Chen and Tsi, 1984).

However, genus *Paphiopedilum* was suggested as the sister with two American genera *Phragmipedium* and *Mexipedium* according to morphology, plastid *rbc*L (Albert, 1994), ITS (Cox et al., 1997), and both nuclear and plastid genes (Guo et al., 2012). These inferences are conflict to the hypothesis of the divergence between *Paphiopedilum* and *Cypripedium* in China, but implied the divergence of *Paphiopedilum* from the group of *Phragmipedium* + *Mexipedium*, by which the slipper orchids (Cypripedioideae) were hypothesized widespread throughout North America and Asia in the past (Atwood, 1984; Albert, 1994; Cox et al., 1997).

Subgenus *Parvisepalum* in southwest China and Vietnam diverged earlier from the other subgenera of genus *Paphiopedilum*. The coalescence time of subgenera *Parvisepalum*, *Brachypetalum,* and *Paphiopedilum* were tracking back to the Upper Miocene (Guo et al., 2015). Subgenus *Brachypetalum* in mainland Southeast Asia was descended from the subgenus *Parvisepalum* inferred by S-DIVA (**Figure 5**), which agrees with other disjunctions at the Southern China and Indochina (Guo et al., 2015) or Sunda Shelf and New Guinea/Australia (Tougard, 2001; Lohman et al., 2011; Tsai et al., 2015).

**Figure 6:**
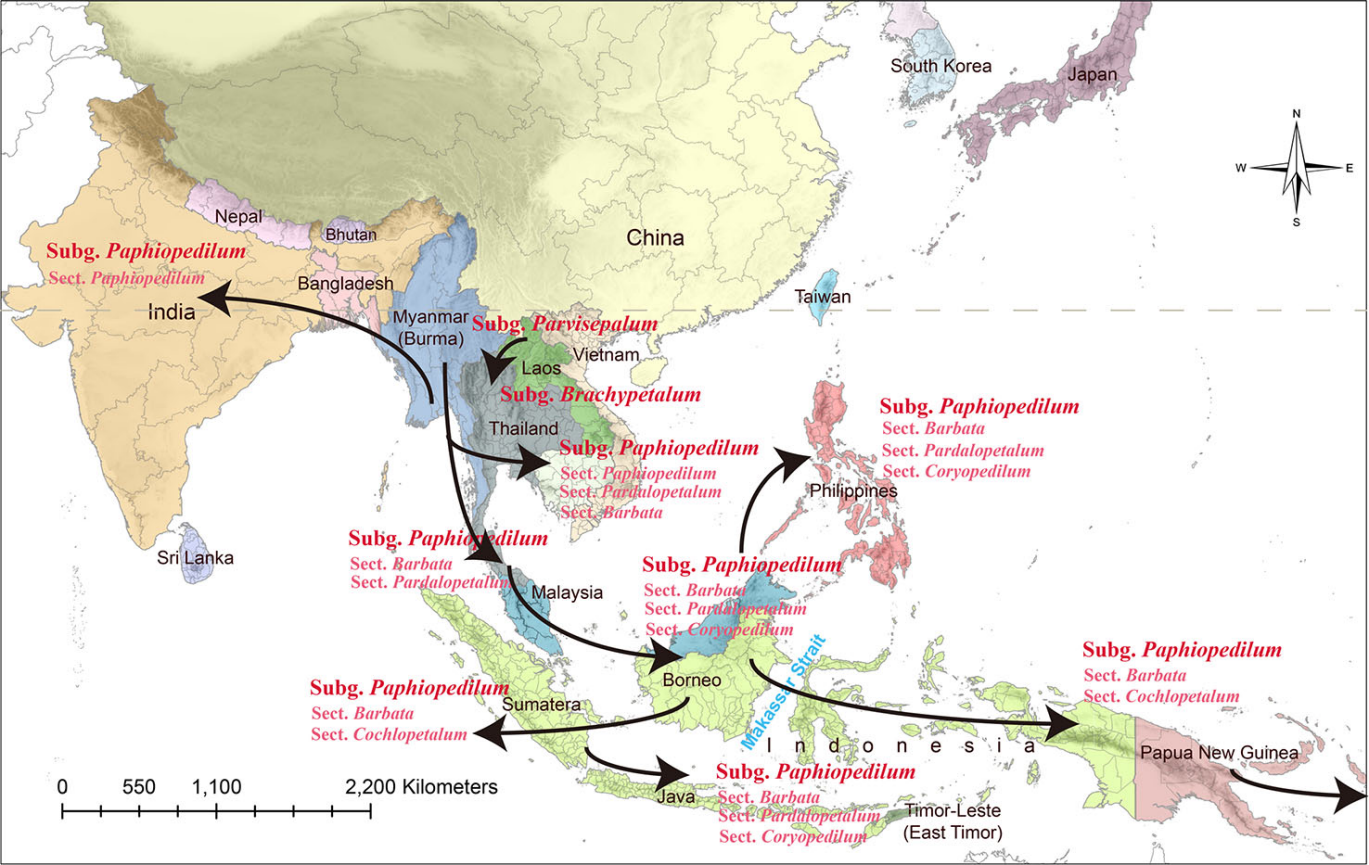
The possible evolutionary routes of the genus *Paphiopedilum*.

The subgenus *Paphiopedilum* is further descended and evolved quickly in the Sunda Shelf. A land bridge might connect Mindoro, Palawan, Borneo, the Malay Peninsula, Borneo, Sumatra, Java, Bali, and various parts of the Philippines when sea levels falling during Pleistocene (about 0.01~1.8 Mya) (van Oosterzee, 1997) (**Figure 1**). Surfaced land bridge connected these regions and was beneficial to the interisland and continent-island dispersal in Southeast and South Asia (van Oosterzee, 1997). The clade of *Coryopedilum* + *Pardalopetalum* was the first derived in subgenus *Paphiopedilum* based on the phylogenetic tree, which reflects in the sympatric distribution of subgenus *Brachypetalum* and section *Pardalopetalum* (**Figure 6**). Following this clade formation, sections *Paphiopedilum* and *Barbata* were subdivided and dispersed throughout Southeast Asian archipelagoes across the land bridge during the glacials. The southward expansion from continental Asia into the Greater Sunda Islands through the Indochina and Malay Peninsulas were also reported in other taxa, e.g., *Lithocarpus* (Fagaceae) (Yang et al., 2018). Such colonization events between continental Asia and the Greater Sunda Islands mostly occurred during Miocene and Plio-Pleistocene (de Bruyn et al., 2014). As a “corridor,” Indochina reveals high flora diversity and the high species richness, which facilitates the *in situ* speciation (de Bruyn et al., 2014).

Another flora diversity hotspot is Borneo (de Bruyn et al., 2014), which is also important for the genus *Paphiopedilum*. The section *Cochlopetalum*, which is found only in Sumatra and Java, might represent a group derived from Borneo. The S-DIVA inferred multiple times dispersal events sourced from Borneo with two vicariances to illustrate the current distribution of the five sections within subgenus *Paphiopedilum* (**Figure 5** and **Table 2**). The Borneo is the second original center of *Paphiopedilum*. The tropical forests and rugged topography harbor diversified niches opened to the speciation of organisms. The repeated submergence and emergence of land bridges could promote repeated genetic isolation and gene flow between closed related taxa. During the Plio-Pleistocene glacial oscillations. This process accelerates the diversification rates in the Sunda Super Islands.

Dispersal and vicariance events that exposed geographic isolation among taxa might be due to the land bridge submergence (Chiang et al., 2009; Chiang et al., 2013; Ge et al., 2012; Ge et al., 2015; Hsu et al., 2013) in Sunda Shelf and Sunda Super Island during the Pleistocene (**Figure 5** and **Table 2**) (Guo et al., 2015; Tsai et al., 2015). Cenozoic collision accompanied by a cyclical climate (glacial oscillations) caused by the fragmentation of the Sunda Super Islands (de Bruyn et al., 2014). The Borneo, Java, Sumatra, and the southern Philippines belong to the Sunda plate, Sulawesi is composed of broken plates, and the Moluccas and New Guinea belong to the Australian plate (Hall, 1998). The deep trenches between these plates cause segregation of species between Sundaland and the islands in the east. Because of the seed germination relies on symbiotic fungi, the geographical isolation maybe not only influences orchid itself but also in symbiotic fungi. However, these inferences still need further verification.

## Conclusions

In summary, the origin and coalescence time of genus *Paphiopedilum* tracked back to Southern China/Eastern Indochina since late Miocene and early Pliocene, while the range expansion and species divergence were related to sea-level fluctuations during the Plio-Pleistocene glacial cycles. Historical geological barriers shaped a pattern of vicariance among disjunct distributed subgenera after isolated ancestral populations. The ancestral taxa of subgenus *Paphiopedilum* migrated from Southern China/Eastern Indochina to south which developed quickly in the Sunda Shelf. Due to the submergence of the Sunda Shelf and Sunda Super Island, species of subgenus *Paphiopedilum* dispersed with isolation between islands as well as subsequent *in situ* speciation within islands from other taxa within section or subgenus, which accelerated species divergence in subgenus *Paphiopedilum*. *Paphiopedilum* distributes in four of 25 biodiversity hotspots (Myers et al., 2000), the Indo-Burma, Sundaland, Wallacea, and Philippines, where are also the “major evolutionary hotspots” (de Bruyn et al., 2014). It is suggests that rich and fascinating historical biogeographic events have created rich species diversity there, such as the case of *Paphiopedilum*. However, deforestation has caused the so-called “empty forest syndrome” (de Bruyn et al., 2014). We hope that these areas will not become extinction hotspots, even though they are almost now.

## Data Availability Statement

The datasets generated for this study are available on request to the corresponding author.

## Author Contributions

Conceived and designed the experiments: C-CT and Y-CC. Performed the experiments: C-CT, P-CL, Y-ZK, C-HC, and Y-CC. Analyzed the data: C-CT, P-CL, Y-ZK, C-HC, and Y-CC. Contributed reagents/materials/analysis tools: C-CT, P-CL, Y-ZK, C-HC, and Y-CC. Wrote the paper: C-CT, P-CL, and Y-CC. Conceived of the study, edited the manuscript, and approved the final manuscript: C-CT, P-CL, Y-ZK, C-HC, and Y-CC.

## Funding

This research was supported by funding from the Ministry of Science and Technology, Taiwan (MOST 105-2621-B-110-003-MY3 and MOST 105-2621-B-110-001) to Y-CC and by partial financing (the Higher Education Sprout Project) of NSYSU.

## Conflict of Interest

The authors declare that the research was conducted in the absence of any commercial or financial relationships that could be construed as a potential conflict of interest.

## Supplementary Material

Supplementary Figure S1 Phylogenetic relationships using Maximum Likelihood resulting from analysis of the combined data matrix (nuclear ribosomal ITS, and trnL-F spacer) from 78 Paphiopedilum and 6 outgroup species.

Supplementary Figure S2 Results of calescence time estimations performed with BEAST 1.8.0 for the from 78 Paphiopedilum taxa and 6 outgroup species based on the combined data matrix (nuclear ribosomal ITS, and trnL-F spacer).

Supplementary Figure S3 Ancestral distributions reconstructed by the Statistical dispersal–vicariance analysis (S-DIVA, Yu et al., 2010) and Bayes–Lagrange Statistical dispersal–extinction–cladogenesis (S-DEC) model (Ree and Smith, 2008) performed in RASP 3.2 (Yu et al., 2015). Phylogenetic relationships of the 78 Paphiopedilum species, plus the six outgroups, obtained from sequence judgments of the combined sequence and generated by BEAST. The distribution areas of extant the 78 Paphiopedilum species species are marked in capitals A–E and I ((A) China, Nepal, India, Bhutan, Burma, Thailand, Malaysia, Cambodia, Vietnam, and Laos; (B) Sumatra, Borneo and Java; (C) Sulawesi and Moluccas; (D) New Guinea; (E) Philippines; and (I) outgroup), respectively. The green and blue circles indicate the vicariance and dispersal events obtained from the RASP analysis, respectively.
