# Supplementary material for: Phylogeny and Historical Biogeography of Paphiopedilum Pfitzer (Orchidaceae) Based on Nuclear and Plastid DNA
Source: Front Plant Sci. 2020 Feb 27;11:126. doi: 10.3389/fpls.2020.00126 (PMC7056885; doi:10.3389/fpls.2020.00126)
Supplement: Supplementary Figure S1 — Phylogenetic relationships using Maximum Likelihood resulting from analysis of the combined data matrix (nuclear ribosomal ITS, and trnL-F spacer) from 78 Paphiopedilum and 6 outgroup species. [file Presentation_1.zip › Files/Supplementary Table S1 and S3.docx]

Supplementary Table S1. PCR primers and PCR reaction conditions used in this work.

| Fragment | Primer sequence (5’-3’) | PCR conditions |
| --- | --- | --- |
| ITS | 5’ TCGTAACAAGGTTTCCGTAGGT 3’  5’ GTAAGTTTCTTCTCCTCCGCT 3’ | The first step: Initial denaturation, 94ºC for 3 min, followed by 10 cycles of denaturation at 94 ºC for 45 s, annealing at 58ºC for 45 s, and extension at 72ºC for 1 min.  The second step: involved 30 cycles of denaturation at 94 ºC for 45 s, annealing at 54 ºC for 45 s, extension at 72 ºC for 1 min, with a final extension for 10 min at 72 ºC. |
| *trn*L intron | c: 5’ CGAAATCGGTAGACGCTACG 3'  d:5’ GGGGATAGAGGGACTTGAAC 3' | The first step: Initial denaturation, 94ºC for 3 min, followed by 10 cycles of denaturation at 94 ºC for 30 s, annealing at 68ºC for 10 s, and extension at 72ºC for 45 s.  The second step: involved 30 cycles of denaturation at 94 ºC for 30 s, annealing at 66 ºC for 10 s, extension at 72 ºC for 45 s, with a final extension for 5 min at 72 ºC. |
| *trn*L-F spacer | e:5’ GGTTCAAGTCCCTCTATCCC 3'  f:5’ ATI'TGAACTGGTGACACGAG 3' | The first step: Initial denaturation, 94ºC for 3 min, followed by 10 cycles of denaturation at 94 ºC for 30 s, annealing at 68ºC for 30 s, and extension at 72ºC for 30 s.  The second step: involved 30 cycles of denaturation at 94 ºC for 30 s, annealing at 66 ºC for 30 s, extension at 72 ºC for 30 s, with a final extension for 5 min at 72 ºC. |
| *atp*B-*rbc*L spacer | 5’ CATCTAGGATTTACATATAC 3'  5’ GTCAATTTGTAATCTTTAAC 3’ | The first step: Initial denaturation, 94ºC for 3 min, followed by 10 cycles of denaturation at 94 ºC for 45 s, annealing at 54ºC for 30 s, and extension at 72ºC for 1 min.  The second step: involved 30 cycles of denaturation at 94 ºC for 45 s, annealing at 52 ºC for 30 s, extension at 72 ºC for 1 min, with a final extension for 5 min at 72 ºC. |

Supplementary Table. S3. The ancestral areas and dispersal – vicariance analysis inferred through RASP from analysis of the combined data matrix (nuclear ribosomal ITS, and *trnL-F* spacer) from 78 *Paphiopedilum* and 6 outgroup species. Ancestral areas for the node and the number of dispersal (Dis), vicariance (Vic) and extinction (Ext) events is shown.

| Node | Ancestral areas | RASP ROUTE | Dis | Vic | Ext | Prob |
| --- | --- | --- | --- | --- | --- | --- |
| 167 | [F] | F->F^F->F\|F | 0 | 0 | 0 | 1.00 |
| 166 | [F] | F->F^F->F\|F | 0 | 0 | 0 | 1.00 |
| 164 | [F] | F->F^F->FA^F->F\|AF | 1 | 0 | 0 | 1.00 |
| 163 | [AF] | AF->F\|A | 0 | 1 | 0 | 1.00 |
| 161 | [A] | A->A^A->AB^A->A\|AB | 1 | 0 | 0 | 0.50 |
| 160 | [A] | A->A^A->A\|A | 0 | 0 | 0 | 1.00 |
| 152 | [AB\|A] | AB->AB^A->ABE^A->A\|ABE | 2 | 0 | 0 | 1.00 |
| 150 | [A] | A->A^A->A\|A | 0 | 0 | 0 | 1.00 |
| 147 | [A] | A->A^A->A\|A | 0 | 0 | 0 | 1.00 |
| 132 | [ACE\|ABE\|ABCE\|BCE\|BE\|AB\|B\|ABC\|AC] | ACE->ACE^B->ABEC^B->ABE\|BC | 2 | 1 | 0 | 0.02 |
| 106 | [B] | B->B^B->B\|B | 0 | 0 | 0 | 1.00 |
| 105 | [B] | B->B^B->B\|B | 0 | 0 | 0 | 1.00 |
| 101 | [B] | B->B^B->BCE^B->BCE\|B | 2 | 0 | 0 | 1.00 |
| 88 | [ABC\|ABE\|ABCE\|A\|AB\|AC] | ABC->AC->AC^A->ACE^A->A\|ACE | 2 | 0 | 1 | 0.02 |
