# Supplementary figures and images for: Phylogeny and Historical Biogeography of Paphiopedilum Pfitzer (Orchidaceae) Based on Nuclear and Plastid DNA
Source: Front Plant Sci. 2020 Feb 27;11:126. doi: 10.3389/fpls.2020.00126 (PMC7056885; doi:10.3389/fpls.2020.00126)

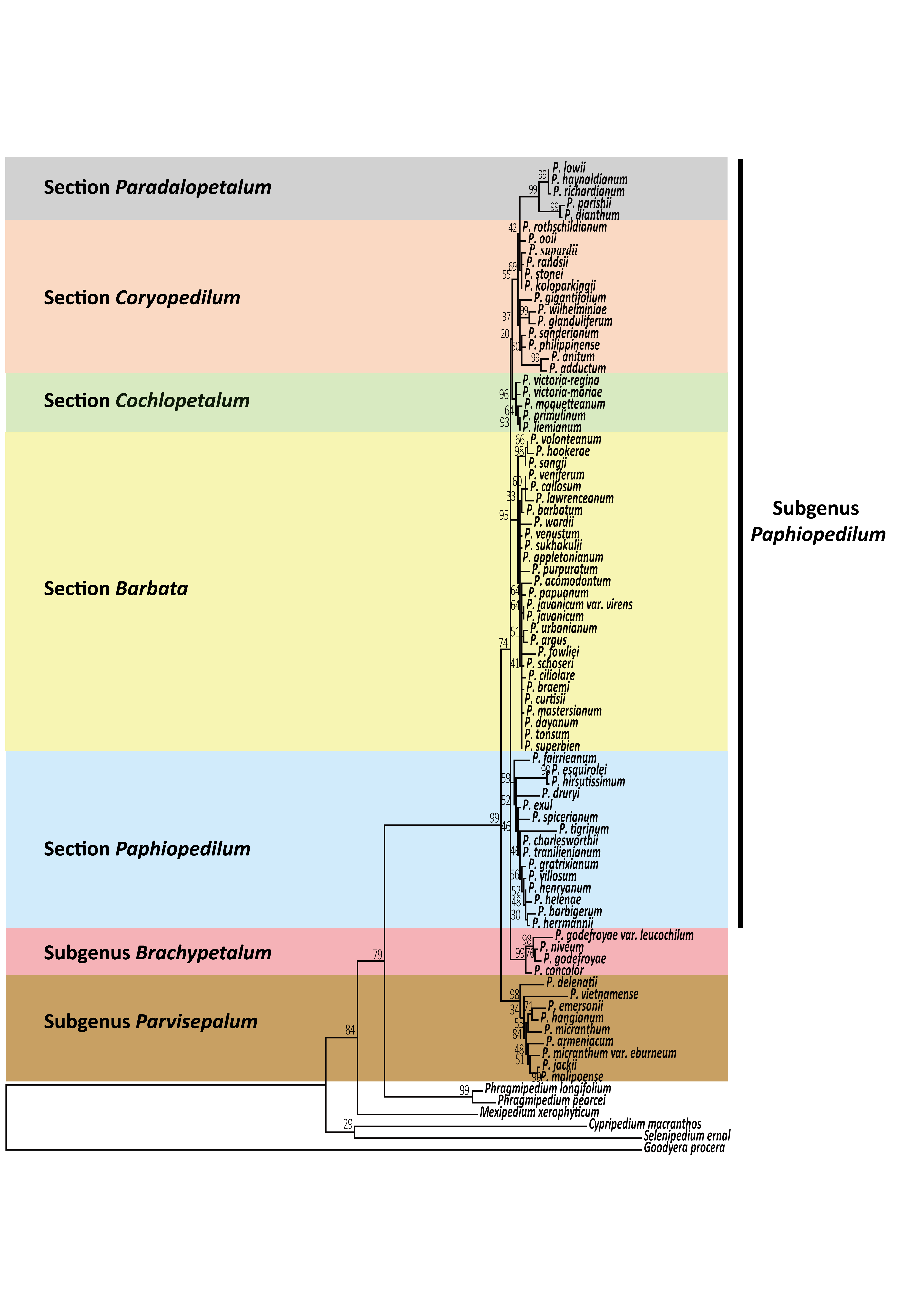

Supplement: Supplementary Figure S1 — Phylogenetic relationships using Maximum Likelihood resulting from analysis of the combined data matrix (nuclear ribosomal ITS, and trnL-F spacer) from 78 Paphiopedilum and 6 outgroup species. [file Presentation_1.zip › Files/Supplementary Figure S1.TIF]

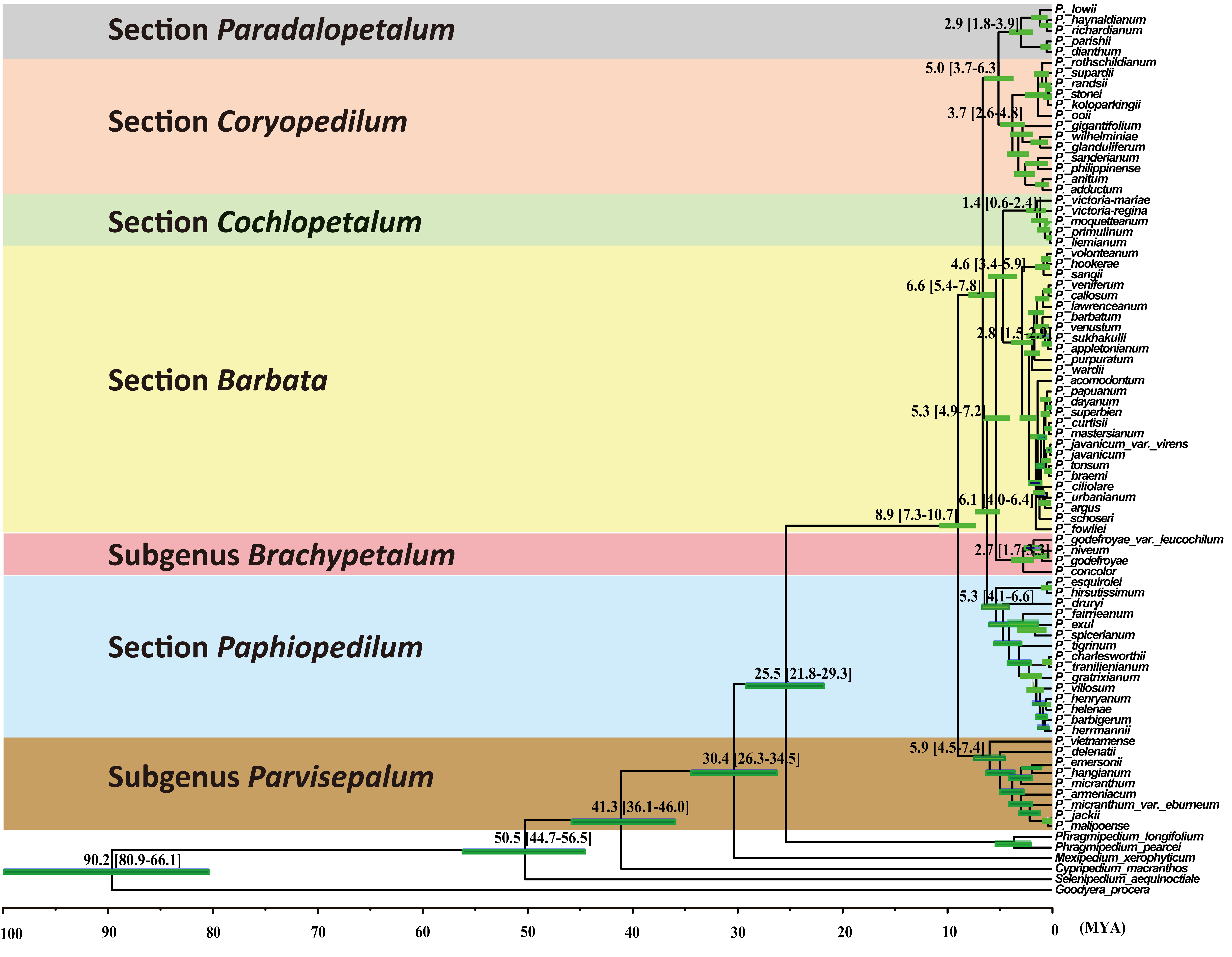

Supplement: Supplementary Figure S1 — Phylogenetic relationships using Maximum Likelihood resulting from analysis of the combined data matrix (nuclear ribosomal ITS, and trnL-F spacer) from 78 Paphiopedilum and 6 outgroup species. [file Presentation_1.zip › Files/Supplementary Figure S2.TIF]

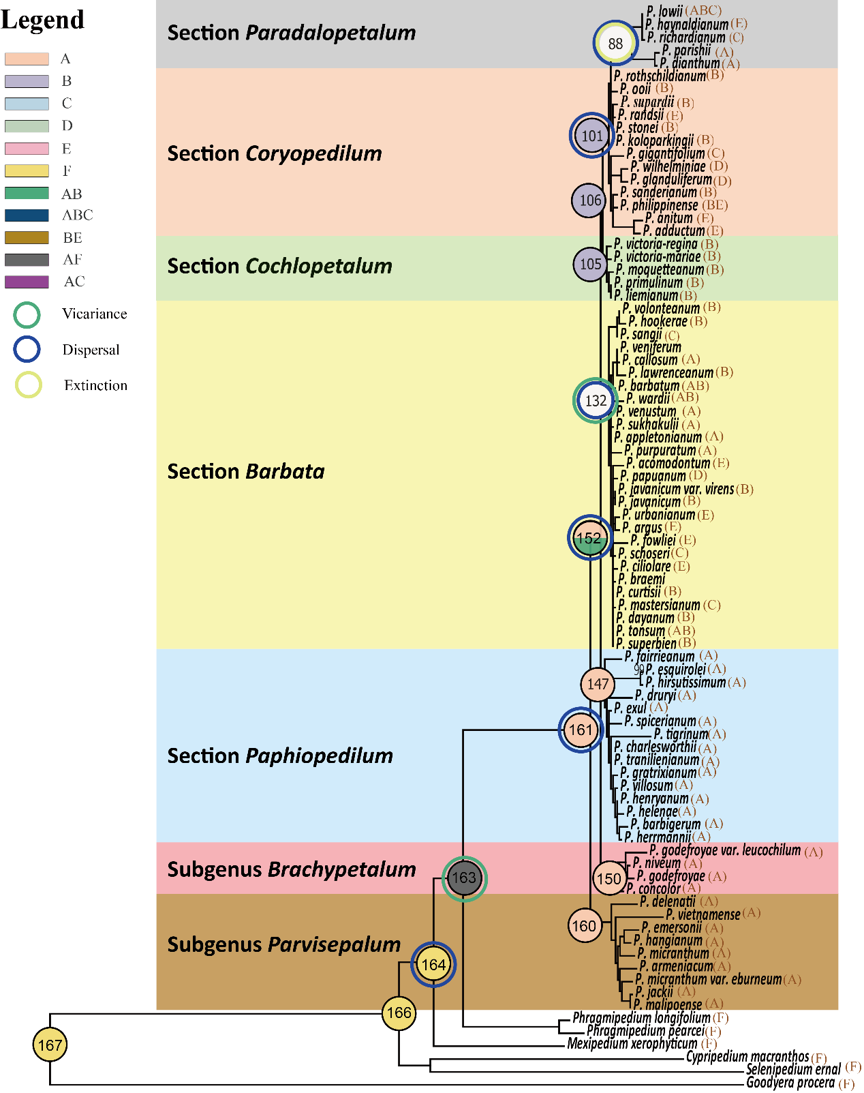

Supplement: Supplementary Figure S1 — Phylogenetic relationships using Maximum Likelihood resulting from analysis of the combined data matrix (nuclear ribosomal ITS, and trnL-F spacer) from 78 Paphiopedilum and 6 outgroup species. [file Presentation_1.zip › Files/Supplementary Figure S3.png]
